# Supplementary material for: Natural Inspired Carboxymethyl Cellulose (CMC) Doped with Ammonium Carbonate (AC) as Biopolymer Electrolyte
Source: Polymers (Basel). 2020 Oct 26;12(11):2487. doi: 10.3390/polym12112487 (PMC7693293; doi:10.3390/polym12112487)
Supplement: Supplementary file 1 [file polymers-12-02487-s001.pdf]

polymers

Supplementary Material:

Natural Inspired Carboxymethyl Cellulose (CMC) Doped with Ammonium Carbonate (AC) as Biopolymer Electrolyte

M.I.H. Sohaimy [1] and M.I.N. Isa [1, 2,*]

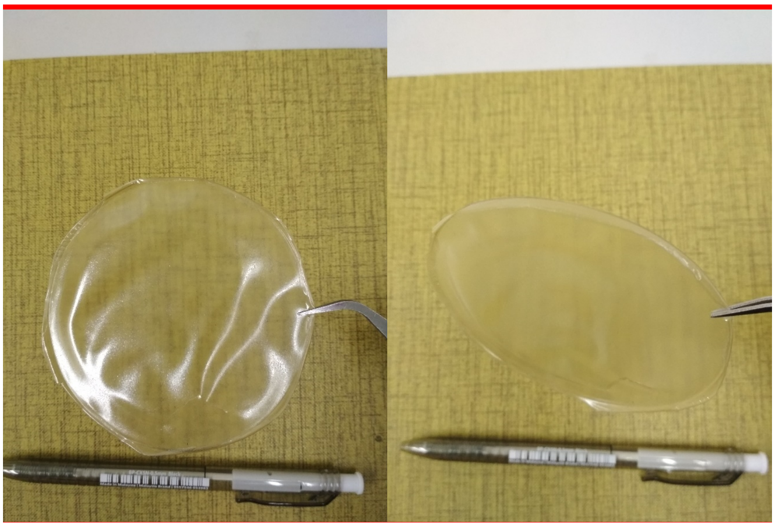

Figure S1. The physical appearance of CMC-AC biopolymer films.

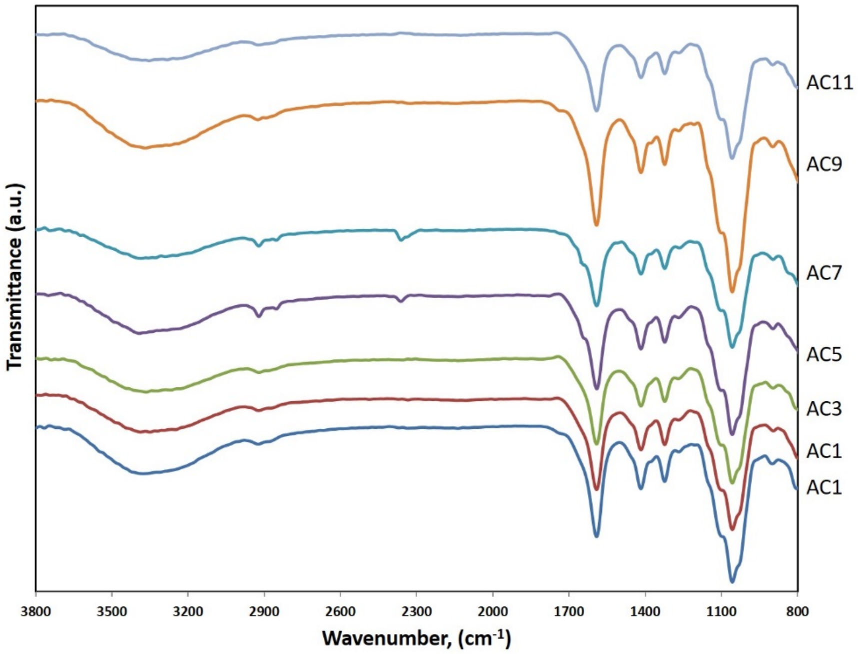

Figure S2. Overall FTIR spectrum of CMC-AC electrolyte.

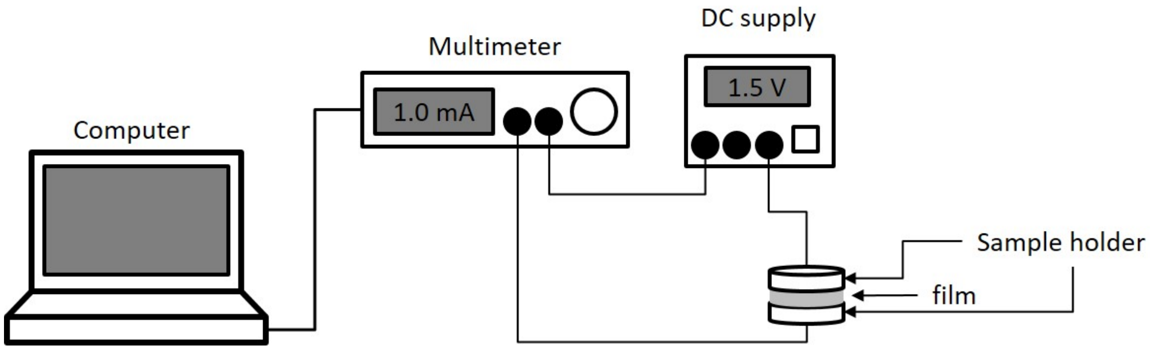

Figure S3. Transference measurement testing setup.

© 2020 by the authors. Submitted for possible open access publication under the terms and conditions of the Creative Commons Attribution (CC BY) license (http://creativecommons.org/licenses/by/4.0/).
